# A randomized double blind placebo controlled trial to assess the safety and efficacy of a patented fenugreek (*Trigonella foenum-graecum*) seed extract in Type 2 diabetics

**DOI:** 10.29219/fnr.v68.10667

**Published:** 2024-06-03

**Authors:** Rajinder Singh Gupta, Amarjit Singh Grover, Pawan Kumar, Apurva Goel, Samudra P. Banik, Sanjoy Chakraborty, Mehul Rungta, Manashi Bagchi, Partha Pal, Debasis Bagchi

**Affiliations:** 1 Department of Medicine, Gian Sagar Medical College & Hospital, Banur, Patiala, India; 2 Department of Surgery, Gian Sagar Medical College & Hospital, Banur, Patiala, India; 3 R&D Department, Chemical Resources (CHERESO), Panchkula, Haryana, India; 4 Regulatory Department, Chemical Resources (CHERESO), Panchkula, Haryana, India; 5 Department of Microbiology, Maulana Azad College, Kolkata, India; 6 Department of Biological Sciences, New York City College of Technology/CUNY, Brooklyn, NY, USA; 7 Department of R&D, Dr. Herbs LLC, Concord, CA, USA; 8 Department of Statistics, Maulana Azad College, Kolkata, India; 9 Department of Biology, College of Arts and Sciences, and Department of Psychology, Gordon F. Derner School of Psychology, Adelphi University, Garden City, NY, USA

**Keywords:** Fenugreek (Trigonella foenum-graecum) seeds, T2D, Clinical Investigation, Fasting Glucose, Post-Prandial Glucose, HbA1c, Antihyperglycemic therapeutic

## Abstract

**Background:**

Fenugreek plant (*Trigonella foenum-graecum*) constitutes a traditionally acclaimed herbal remedy for many human ailments including diabetes, obesity, neurodegenerative diseases, and reproductive disorders. It is also used as an effective anti-oxidative, anti-inflammatory, antibacterial, and anti-fungal agent. The seed of the plant is especially enriched in several bioactive molecules including polyphenols, saponins, alkaloids, and flavonoids and has demonstrated potential to act as an antidiabetic phytotherapeutic. A novel patented formulation (Fenfuro^®^) was developed in our laboratory from the fenugreek seeds which contained >45% furostanolic saponins (HPLC).

**Objective:**

A placebo-controlled clinical compliance study was designed to assess the effects of complementing Fenfuro^®^ on a randomized group of human volunteers on antidiabetic therapy (Metformin and sulphonylurea) in controlling the glycemic index along with simultaneous safety assessment.

**Study methodology and trial design:**

In a randomized double-blind, placebo-controlled trial, 42 individuals (21 male and 21 female volunteers) in the treatment group (out of 57 enrolled) and 39 individuals (17 male and 22 female volunteers) in the placebo group (out of 47 enrolled), all on antidiabetic therapy with Metformin/Metformin with sulphonyl urea within the age group of 18–65 years were administered either 1,000 mg (500 mg × 2) (Fenfuro^®^) capsules or placebo over a period of 12 consecutive weeks. Fasting and postprandial glucose along with glycated hemoglobin were determined as primary outcomes to assess the antidiabetic potential of the formulation. Moreover, in order to evaluate the safety of the formulation, C-peptide and Thyroid Stimulating Hormone (TSH) levels as well as immunohematological parameters were assessed between the treatment and placebo groups at the completion of the study.

**Results:**

After 12 weeks of administration, both fasting as well as postprandial serum glucose levels decreased by 38 and 44% respectively in the treatment group. Simultaneously, a significant reduction in glycated hemoglobin by about 34.7% was also noted. The formulation did not have any adverse effect on the study subjects as there was no significant change in C- peptide level and TSH level; liver, kidney, and cardiovascular function was also found to be normal as assessed by serum levels of key immunohematological parameters. No adverse events were reported.

**Conclusion:**

This clinical compliance study re-instated and established the safety and efficacy of Fenfuro^®^ as an effective phytotherapeutic to treat hyperglycemia.

## Popular scientific summary

- Antihyperglycemic potential of a novel Fenugreek seed extract with >45% furostanolic saponins was investigated in a randomized double blind placebo controlled trial.
- Administration of a daily dosage of 500 mg × 2 for 12 weeks resulted in significant decrease in fasting and post-prandial glucose as well as glycated hemoglobin.
- There was no adverse effect as revealed by C peptide analysis, TSH levels, as well as other immunohematological parameters.
- The studies re-established the efficacy of Fenugreek in the treatment of hyperglycemia.

Lifestyle disorders have emerged as the second most ominous threat to human health after cancer. Increasing stress, consumption of junk food, and lack of physical activity have together culminated into chronic and critical ailments such as Type 2 diabetes (T2D), obesity, and consequent cardiovascular disorders. In particular, the incidences of T2D have increased unprecedentedly and more than half of the global population has been projected to be affected by the end of 2045 (1–6). In obese individuals or in cases of extreme lipoatrophy, insufficiency of storage space results in ectopic fat deposition, especially around the belly and hip joint.

Adiponectin is a cytokine secreted by adipose tissues that contributes to the regulation of glucose homeostasis by dampening gluconeogenesis and increasing glycolysis and β-oxidation (7–9). Under conditions of increased fat deposition and dyslipidemia, adiponectin levels are lowered substantially (10), which eventually leads to insulin resistance and disposition to T2DM. In addition, recent evidence has suggested that dysbiosis of the gut microbiome is also a major factor for development of insulin resistance (11).

Despite unprecedented progress in medical science, there is currently no FDA-approved therapy for the prevention of insulin resistance and a handful of antidiabetic drugs have achieved FDA approval for controlling hyperglycemia. As per recommendations of the Diabetes Prevention Program and its Outcomes Study (DPPOS), a controlled diet regimen with restricted carbohydrate intake and consumption of food with low sodium and low glycemic index is the need of the hour to arrest dispositions to Type 2 diabetes (12). Since one of the significant hallmarks of the shift of pre-diabetes to T2DM is the functional dysbiosis of the beta cells, the current line of drugs has been designed to augment the ability of the β-cells to secrete insulin. The sulphonyl urea-based drugs promote the release of insulin by inhibiting the action of sulfonylurea receptor-1 (SUR-1), the regulatory sub-unit of the K+ channel in β-cells (13). The glucagon-like peptide-1 (GLP-1), a hormone produced by L cells found along the lining of the GI epithelium is responsible for simultaneously stimulating the secretion of insulin and inhibiting glucagon release by pancreatic α-cells. Owing to the low biological half-life of GLP-1, use of agonists such as dipeptidyl peptide-4 (DPP-4) inhibitors has been in vogue for the treatment of T2DM (14). However, these drugs are primarily meant for boosting insulin production instead of increasing insulin sensitivity and therefore are less likely to act as long-term cures of T2DM.

The second mechanism of treatment for T2DM is constituted by the drugs that improve insulin sensitivity instead of boosting insulin release. Thiazolidinediones (TZDs) are a class of drugs meant for increasing insulin sensitivity in skeletal muscle, liver, and adipose tissue. They also aid in the management of obesity by affecting transport of fat from liver and skeletal muscles to adipocyte redistribution through activation of nuclear receptor peroxisome proliferator-activated receptor gamma (PPARγ) (15). One of the more commonly recommended medicines for augmenting insulin sensitivity is metformin, a biguanide class of antihyperglycemic agent which acts in the peripheral tissues by inhibiting gluconeogenesis and thus, decreasing the endogenous hepatic output of glucose.

Metformin exerts its effect both by Adenosine Monophosphate Kinase (AMPK) dependent and AMPK independent mechanisms of mitochondrial respiration arrest. A third class of drugs are alpha-glucosidase inhibitors which block the GI tract hydrolases to delay absorption of sugars and thereby decrease postprandial blood glucose and insulin levels (16). Another line of therapy includes the use of sodium-glucose cotransporter 2 (SGLT2) which lowers blood sugar levels by inhibition of renal glucose reabsorption (17).

Over the years, a significant concern has been raised over the use of the above listed compounds as anti-diabetics due to their parallel intervention in several cellular signaling pathways involved in the maintenance of metabolic homeostasis. The use of metformin has been reported to be associated with diarrhea and lactic acidosis (18) whereas sulphonyl urea has been reported to perturb the physiological activity of the thyroid gland, cause dysbiosis in lipid metabolism, and also result in hypoglycemia in a few instances (13). Keeping in mind the safety concerns associated with the use of these allopathic drugs, an increasing number of people are resorting to the use of Ayurveda or herbal dietary bioactives for effective management of T2DM (19, 20) Amongst the plethora of dietary bioactives used for the treatment of Type 2 diabetes, the most effective ones are the diverse group of polyphenols including ellagic acid (21), resveratrol (22), emodin (23), curcumin (24), baicalein (25), genistein (26), Kaempferol (27), Quercetin (28, 29), and several others. In this respect, the fenugreek plant (*Trigonella foenum-graecum*) has been used traditionally as a significant source of anti-glycemic phytotherapeutic to arrest the progression of prediabetic patients to diabetes.

**Fig. 1:**
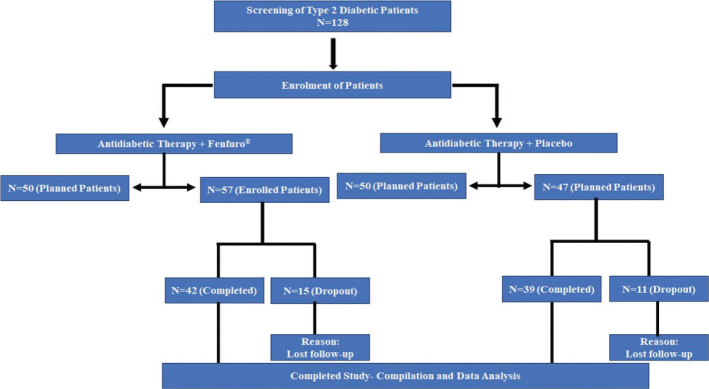
Major furostanolic saponins from *Trigonella foenum-graecum* with biological effector functions: Saponins are classified according to the aglycone moieties and the attached carbohydrate. The structures were taken from ChemSpider and redrawn using ChemDraw and BioRender.

The seeds and leaves of fenugreek plant have been used traditionally especially in Africa, the Indian Subcontinent, and China to address diverse ailments and disorders including obesity, oxidative stress, loss of metabolic homeostasis, and dyslipidemia (30). The pharmacotherapeutic potential of fenugreek is attributed to its strong bioactive component reservoir including steroids, polyphenols, alkaloids, saponins, hydrocarbons, galactomannan fiber etc. The plant has significant reserves of furostanolic saponins like trigoneoside, isoorientin, orientin, vitexin, and isovitexin (31, 32) which forms the basis of biological effector functions of this plant (Fig. 1). The components of fenugreek act in a synergistic manner to lower blood glucose levels by overexpression of glucose transporter (GLUT-2) receptor and sterol regulatory element-binding protein (SREBP1C) mRNA levels (31). The hypoglycemic potential of Fenugreek has been demonstrated earlier in animal models as well in human clinical trials (33, 34). In one such study in a streptazocin-induced diabetic mouse model, administration of fenugreek seed extract at a dose of 100 mg/kg of body weight resulted in parallel decrease in urea, creatinine, AST, ALT, and triglycerides in addition to significant reductions in blood glucose (35). A novel patented fenugreek seed extract composition (Fenfuro^®^) was developed in our laboratory which increased the efficacy of Metformin (36). The extraction procedure resulted in retention of a significant amount of furostanolic saponins and in a subsequent placebo-controlled study, was shown to decrease postprandial glucose by more than 30% (37). The present studies were conducted as a follow-up of the earlier reports to test the efficacy of supplementation with Fenfuro^®^ in a study cohort on antidiabetic therapy on levels of fasting and post-prandial glucose with simultaneous evaluation of its safety with regard to liver function and other hematological parameters (37).

## Materials and methods

### Standardized *Trigonella foenum-graecum* seed extract (Fenfuro^®^)

A patented *T. foenum-graecum* seed extract (Fenfuro^®^, > 45% furostanolic saponins (HPLC)); Batch Nos. FEN0615; Mfg date June 2015; and FEN0715; Mfg date July 2015; Patents 8,217,165B2 (US); EP 2285821B1 and EP2437763 B1 (European Patents); 285647 and 371174 (Indian patents); AP 3077 (African Patent); and Patents CN102448479 B (Chinese Patent) was manufactured using a novel water–ethanol extraction process in a GMP-NSF certified plant.

### HPLC of Fenfuro^®^

In order to estimate the furostabolic saponin content in Fenfuro^®^, 50 mg of dry powdered extract was dissolved in 50 mL of acetonitrile:water (1:3) diluent phase, sonicated for 15 min, and subsequently filtered through 0.45 μm syringe filter. HPLC was conducted with 20 μL aliquot of the sample in gradient elution mode (acetonitrile:water::1:9) using a Nucleosil 5 μ C18 (2) column 250 × 4.6 mn at a flow rate of 1 mL/min and detection at 205 nm (Column oven temp set at 30°C).

Subsequently, the content of furostanolic saponin present was ascertained using the standard protodioscin run under exactly same conditions according to the method given below


$$\begin{array}{l} \text{Protodioscin = }\frac{\text{Area of Protodioscin}}{\text{Standard Area of Protodioscin}}\\ \text{x}\frac{\text{Concentration of standard}}{\text{Concentration of test}}\text{x Purity of standard}\\ \text{Total Saponins = }\frac{\text{Area of all saponin peaks}}{\text{Standard area of protodioscin}}\\ \text{x}\frac{\text{Concentration of standard}}{\text{Concentration of test}}\text{x Purity of standard} \end{array}$$

### Ethical conduct and regulatory approvals

The medical and clinical procedures of this randomized single-center, placebo-controlled double- blind study were performed in compliance and accordance with the International Council for Harmonization (ICH) guidelines for Good Clinical Practices (GCP), while all essential clinical documents were maintained and stored as per International Ethical Standards guaranteed by the Declaration of Helsinki and its subsequent amendments. Subject confidentiality was strictly maintained. All subjects were recruited as per the inclusion and exclusion criteria approved by the Institutional Review Board (IRB) and subject confidentiality was strictly maintained.

This study obtained the Institutional Ethics Committee’s (IEC) approval of the Gian Sagar Medical College, Village Ram Nagar, Rajpura, Patiala (Protocol #CR-FEN/PREDIA/02/15) dated Nov 23, 2015, Ref: IRB Proposal “Clinical Evaluation of Fenfuro^®^ (Fenugreek Seed Extract) in Type-2 Diabetic Subjects – An Add-On Study”. Ethics Committee Registration # ECR/572/Inst/PB/2014 was also issued by the Office of Drugs Controller General (India), Directorate of Health Services (New Delhi, India) under Rule 122DD of the Drugs & Cosmetic Rules 1945, India (File #ECR/856/Gian/Inst/PB/2013). This trial was also duly registered on clinicaltrials.gov (NCT03066089).

### Subject consent

All subjects were recruited as per the IEC-approved inclusion and exclusion criteria. All recruited subjects read, understood, and duly signed the IEC-approved health questionnaire and consent forms. Adverse event monitoring was strictly ascertained.

### Project compliance

Study coordinators distributed the placebo and treatment capsules (Fenfuro^®^) to the recruited subjects, while individual data entry was endorsed by the Principal Investigator (PI). Moreover, PI regularly signed off the investigational product (IP) accountability log.

#### Project confidentiality

To minimize or prevent selection bias in the randomized, placebo-controlled investigation, allocation concealment was conducted on the enrolled study participants, investigators, and study coordinators responsible for the assessment of the enrolled subjects entering the trial. Each capsule contained 500 mg of either placebo or Fenfuro^®^ (500 mg) (investigational product) and the capsules were advised to be taken orally as BD dosage. The capsules were opaque and were sequentially numbered in coded packs of 60 capsules in sealed aluminum pouches and used for concealing the identity.

#### Project discontinuation criteria

In case of serious adverse events (as outlined in the safety assessment clause), the norms for ‘stopping’ of trial or ‘discontinuation criteria’ were applicable.

### Subject recruitment

**Fig. 2:**
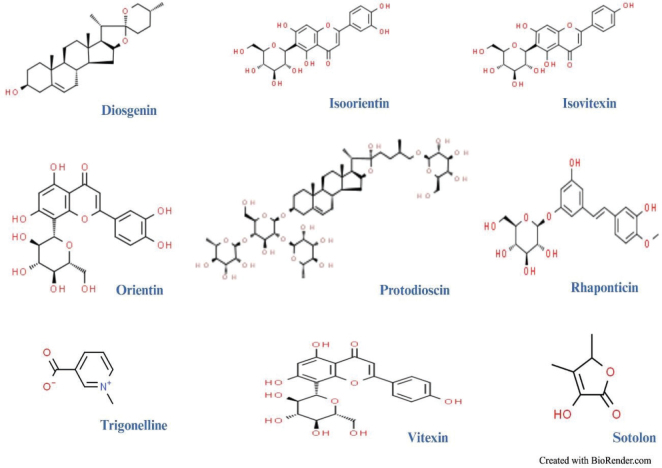
Consort figure showing the number of patients recruited in both treatment (Fenfuro + antidiabetic therapy) and placebo (antidiabetic therapy alone) groups, number of patients who completed the study and workflow.

Recruited subjects were clinically examined, screened, and evaluated using an IRB-approved inclusion and exclusion criteria (Table 1). A total of 128 patients on anti-diabetic therapy (using Metformin or Sulphonylurea or both were screened and a total of 104 male and female subjects were recruited, randomized, and divided into placebo (47 subjects) and Fenfuro^®^ (57 subjects) groups as per the Consort diagram (Fig. 2). Randomization codes were generated by the SAS procedure PROC PLAN using block design. All the recruited subjects were advised not to change their regular physical activities. The demographic data of the placebo and Fenfuro^®^ treated subjects are shown in Table 2. At the completion of the clinical investigation, 39 subjects in the placebo group and 42 subjects in the Fenfuro^®^ group completed the study.

**Table 1:** Inclusion and exclusion criteria

Inclusion criteria
Agrees to written informed consent.
Male and female subjects (18–65 years)
Patient on anti-diabetic therapy
HbA1c level more than 7.5%
Fasting plasma glucose level < 180 mg/dL
Not receiving any steroids
Exclusion criteria
1. Uncooperative subjects
2. Diabetes other than type 2 diabetes mellitus
3. History of any hemoglobinopathy that may affect the determination of glycosylated Hemoglobin (HbA1c)
4. Evidence of renal and liver diseases
5. Lactating or pregnant or planning to conceive females.
6. Physically/mentally unwell as certified by physician-in-charge
7. Participation in any other clinical trial within the last 30 days
8. Subjects with allergy to investigational product

**Table 2:** Demographic data

Parameters	Baseline		*P*-value between group	Completion of 12-weeks of treatment		*P*-value between group
	Placebo	Fenfuro^®^		Placebo	Fenfuro^®^	
Body Weight (kg)	73.00 ± 12.24	74.47 ± 12.18	0.590	72.34 ± 12.26	74.18 ± 12.01	0.499
BMI (kg/m^2^)	27.08 ± 3.43	28.36 ± 3.80	0.117	26.74 ± 3.44	28.20 ± 3.67	0.071
Waist circumference (inches)	46.74 ± 13.36	38.76 ± 3.66	0.0001*	40.07 ± 8.29	38.09 ± 3.41	0.158
Age (years)	50.69	52.45	-	-	-	-
Height (cm)	163.74 ± 12.13 cm	161.86 ± 13.6 cm	-	-	-	-
Pulse (per min)	73.16 ± 6.52	81.71 ± 7.83	-	-	-	-
Sample size	39 (Male 17: Female 22)	42 (Male 21: Female 21)	-	-	-	-
Blood pressure (mmHg)	Diastolic 77.43 ± 6.2; Systolic 125.64 ± 8.4	Diastolic 84.9 ± 6.2 Systolic 129.04 ± 9.97	-	-	-	-

* Indicates statistically significant.

All subjects were advised to take either placebo or Fenfuro^®^ capsules in addition to the Metformin/Sulphonylurea. Both placebo and treatment capsules were advised to take placebo or Fenfuro^®^ capsules (2 capsules of 500 mg each/day) orally over a duration of 12 consecutive weeks. All subjects maintained daily diaries, which were regularly endorsed by the study coordinators and the principal investigator. Adverse event monitoring was routinely conducted.

### Study compliance

Both placebo and Fenfuro^®^ capsules were stored at room temperature in a cool, dry, and dark environment and kept away from direct sunlight. Principal investigator (PI) and study coordinators distributed the placebo and Fenfuro^®^ capsules to all enrolled subjects. The investigational product was allocated and distributed by the site staff only. All enrolled subjects received a Daily diary for recording the details of capsule intake, food intake, daily activities, and any adverse or untoward events. Moreover, all subjects received one pack of 60 capsules in a sealed aluminum pouch at the beginning of each month and were advised to take two capsules each day. The enrolled subjects were advised to record in their daily diaries and get their entries endorsed by the study coordinators and PI. Capsule distribution record was maintained in the IP accountability log provided by the sponsor to the PI, while each entry was recorded individually with the date/signature of the PI and study coordinators. During the technical audit, an IP accountability log was made available by the PI.

### Concomitant medication

The study participants routinely maintained the records of intake of all concomitant prescription medications, including over-the-counter medications (OTC) and non-prescription medications during the investigation and all these were recorded on the case report forms (CRFs).

### Safety assessment

Safety of patients consuming investigational products was routinely and strictly ascertained. Enrolled participants routinely visited the clinic every 4 weeks for regular physical check-ups, returned the unused capsules, and received capsules for the following 4 weeks. Physical health was assessed by checking physical signs of any adverse drug reaction. At baseline and last follow-up visit, the safety was assessed by laboratory investigations at baseline and last follow-up visit.

Furthermore, detailed blood chemistry analyses including serum glutamic pyruvic transaminase activity (SGPT), serum glutamic oxaloacetic transaminase activity (SGOT), serum alkaline phosphatase activity (ALP), serum bilirubin, blood urea nitrogen (BUN) level, serum creatinine level, hemoglobin level, total leukocyte counts (TLC), serum urea, creatinine, cholesterol, LDL-C, HDL-C, and triglyceride levels were performed at the initiation and completion of 12 consecutive weeks of supplementation.

### Efficacy assessment

Subjects suffering from type 2 diabetes for not more than a duration of 5 years were enrolled in this clinical investigation. Furthermore, the HbA1c levels in all the recruited volunteers are >7.5% and the fasting blood glucose are <180 mg/dL. As indicated earlier, all the recruited patients in both the placebo and Fenfuro^®^ groups were on metformin and/or sulfonylurea.

### Fasting glucose (mg/dL) and postprandial glucose (mg/dL) levels

Fasting and postprandial plasma glucose levels (mg/dL) were determined at 0, and 12 weeks of investigation using assay kits from authorized distributor of Johnson & Johnson Ltd, Mumbai, India.

### Glycated Hemoglobin (HbA1c) assessment

Glycated hemoglobin (HbA1c) levels were assessed at the initiation (0 weeks), and completion (12 weeks) of supplementation using kits manufactured by Bio-Rad Laboratories (Irvine, CA, USA).

### Assessment of c-peptide level (ng/mL)

Fasting and postprandial C-peptide levels were measured at 0 and 12 weeks of supplementation using the kits manufactured by DiaSorin S.p.A (Saluggia, Italy).

### Adverse events

Subjects were instructed to document all types of adverse events in their daily diaries during this investigation. All subjects were critically questioned if they had experienced any uncomfortable situation/untoward problems or difficulties during their routine visits to the clinical study center. Altogether, adverse event monitoring was strictly enforced.

### Statistical analyses

Data was described as mean ± standard deviation (SD). The effect of Fenfuro^®^- supplementation was compared with that in the placebo group using t-test. A paired t-test was performed with the data from the study cohort before and after treatment (12 weeks) in case of both the Fenfuro^®^ supplemented group and the placebo group (on anti-diabetic therapy alone). In order to assess the effect of Fenfuro^®^ supplementation, unpaired t-test was performed within the two groups. Null hypothesis was framed as no significant difference before and after treatment for analysis within groups and no significant difference between Fenfuro^®^ supplemented group and placebo group (on antidiabetic therapy); acceptance of alternative hypothesis suggested significant difference in both cases. Both paired and unpaired t-tests were used for primary outcome analysis (fasting and post-prandial blood sugar levels and glycated hemoglobin measurement) whereas only unpaired t-tests were conducted for secondary outcome analysis after the treatment period of 12 weeks (C-peptide, Thyroid Stimulating Hormone [TSH], liver, and kidney function markers and other immune-hematological analyses) to assess the safety of the formulation. F tests were conducted in order to assess any real significant difference between variances within groups.

## Results

### Furostanolic saponin content of Fenfuro^®^

**Fig. 3:**
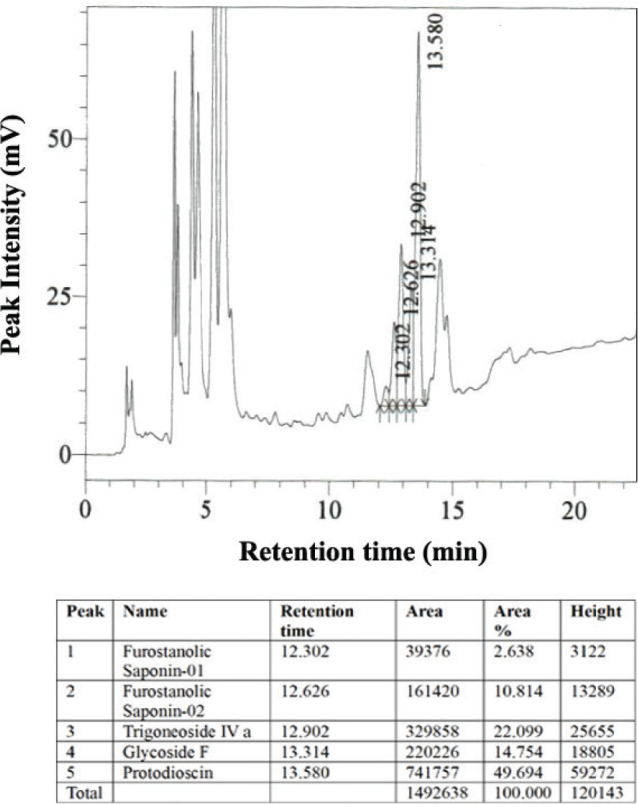
HPLC elution profile of 1 mg/mL Fenfuro^®^ in water: acetonitrile gradient.

The total furostanolic saponin content as ascertained in terms of furostanolic saponin-01, furostanolic saponin-02, Trigoneoside IV and Glycoside F content was found to be more than 45% (Fig. 3).

### Demographic parameters (age and gender wise distribution)

Both male and female subjects participated in this clinical investigation. In the Fenfuro^®^-treated group, the average age of the study population was 52.45 years, with a minimum age of 28 years and a maximum age of 64 years. In the placebo group (on-going anti-diabetic therapy group), the average age of the study population was 50.69 years with a minimum age of 28 years and a maximum age of 65 years. Finally, 46.9% of male and 53.1% of female subjects completed the study. Table 2 displays the demographic data of this investigation.

### Effects of Novel Trigonella foenum graecum (Fenugreek) seed extract, Fenfuro^®^, on fasting and post-prandial plasma glucose levels

**Fig. 4:**
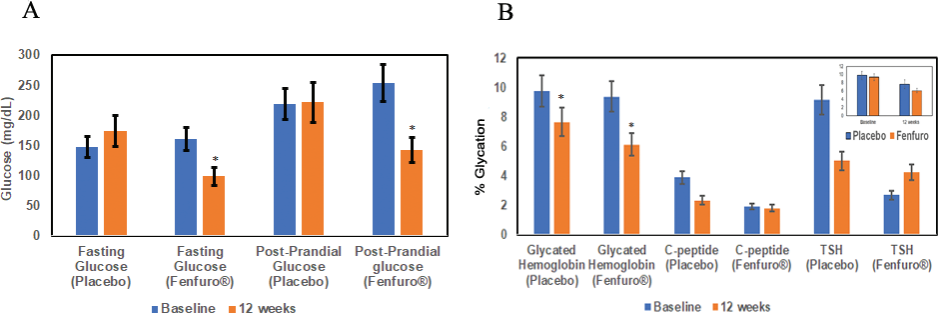
Antidiabetic effect of Fenfuro^®^ supplementation (a) Effect of fasting and post-prandial glucose. (b) Effect on glycated hemoglobin. (c) peptide and TSH. Effects were assessed both within groups (before and after treatment) and between groups (Fenfuro^®^ supplementation and placebo) by paired and unpaired t-test respectively as described in methodology. Significant differences are indicated by stars. A direct comparison between placebo and Fenfuro is also shown in the inset of Fig. 4b.

Effect of supplementation with Fenfuro^®^ on people undergoing antidiabetic therapy was investigated in terms of fasting and post-prandial blood glucose (Fig. 4a, Table 3), In Fenfuro^®^-supplemented subjects, the mean fasting glucose levels decreased significantly (*P* = 0.0001**) from baseline value of 159.97 to 98.76 mg/dL on completion of 12-weeks of treatment, whereas in the placebo group the fasting glucose levels increased from baseline levels of 147.12 mg/dL – 173.76 g/dL (*P* = 0.023*). Alongside, in the Fenfuro^®^-supplemented group, approximately 95.2% of the study population showed a reduction in fasting blood glucose at the completion of 12-weeks of treatment. On the contrary, 60.50% of the study population in the placebo group showed an increase in fasting blood glucose at the completion of 12-weeks of treatment. Overall, in the Fenfuro^®^-supplemented group, a 38.26% decrease in fasting glucose levels was observed, while in the placebo group, a 18.6% increase in the fasting glucose levels was observed at the completion of 12-weeks of treatment. The overall reduction in blood glucose was highly significant in the cohort supplemented with Fenfuro^®^ as compared to the placebo group (on antidiabetic therapy alone). Mean post prandial glucose levels were also reduced significantly (*P* = 0.0001*) in the Fenfuro^®^-supplemented subjects from baseline levels of 254.33 to 142.30 mg/dL at the completion of 12-weeks of treatment, whereas in the placebo group, no significant difference was observed in levels before (219 mg/dL) and after treatment (222 mg/dL). In Fenfuro^®^-treated subjects, a 44.04% reduction in the mean post-prandial glucose levels was observed at the completion of 12 weeks of treatment, while in the placebo group a 1.71% reduction was observed. Moreover, in the Fenfuro^®^-supplemented subjects, approximately 88.10% of the study population showed a reduction in the mean post-prandial glucose levels at the completion of 12-weeks of supplementation, while in the placebo group approximately 47.40% of the subjects showed an increase in the mean post-prandial glucose levels. The reduction in post-prandial glucose was also highly significant in the Fenfuro^®^-supplemented group as compared to the placebo group.

**Table 3:** Effect of Fenfuro^®^ on fasting and post-prandial plasma glucose in the placebo- and Fenfuro^®^-supplemented subjects

Parameters	Groups	Baseline	12-weeks of treatment	*P*-value (within group)	*P*-value (between group)
Fasting glucose (mg/dL)	Placebo	147.12 ± 26.72	173.76 ± 74.53	0.023*	0.019* (Baseline)
	Fenfuro^®^	159.97 ± 21.31	98.76 ± 23.46	0.0001*	0.0001* (Completion)
Post-prandial glucose (mg/dL)	Placebo	219.0 ± 75.55	222.0 ± 103.65	0.0001*	0.055 (Baseline)
	Fenfuro^®^	254.33 ± 86.69	142.30 ± 48.09	0.839	0.0001*

Data are expressed as mean ± SD.

* Significant reduction.

### Effect of Fenfuro^®^ supplementation on Glycated Hemoglobin Level, C peptide level and TSH levels

In Fenfuro^®^-supplemented subjects, mean glycated hemoglobin level (HbA1c) was significantly reduced (*P* = 0.0001*) from the baseline value of 9.37 to 6.11% at the completion of Fenfuro^®^ supplementation over a period of 12 consecutive weeks (Fig. 4b, Table 4). In the placebo group (on-going anti-diabetic therapy) subjects, HbA1c level also significantly reduced from the baseline of 9.78 to 7.65% at the completion of 12-consecutive weeks of treatment. However, mean HbA1c levels decreased by approximately 34.7% in Fenfuro^®^-supplemented subjects, while a reduction of 21.51% was observed in the placebo group. Accordingly, a significantly higher reduction in the HbA1C level was observed in all Fenfuro® supplemented subjects at the completion of 12-consecutive weeks of treatment as compared to the placebo group. Thus, Fenfuro^®^ supplementation improved insulin sensitivity and reduced insulin resistance to a remarkable extent.

**Table 4:** Glycated hemoglobin (HbA1c) levels in the placebo- and Fenfuro^®^-treated subjects

Parameters	Groups	Baseline	12-weeks of treatment	*P*-value (within group)	*P*-value (between group)
% of Glycated Hemoglobin (HbA1c)	Placebo	9.78 ± 1.65	7.65 ± 2.10	0.0001*	0.234 (Baseline)
	Fenfuro^®^	9.37 ± 1.45	6.11 ± 1.30	0.0001*	0.0001* (Completed)

Data are expressed as mean ± SD.

* Significant reduction. A paired t-test was adopted for the analysis within group and unpaired t-test was followed for analysis between group as detailed in methodology.

### Effects of Fenfuro^®^ on mean C-Peptide level and TSH levels

In order to assess the safety of the formulation, C peptide, TSH, and other immunohematological parameters were assessed. No significant changes in mean C-peptide and TSH levels were observed between the placebo and Fenfuro^®^ supplemented groups as compared to the baseline (Table 5).

**Table 5:** Effect of Fenfuro^®^ on mean C-peptide level and Thyroid Stimulating Hormone (TSH) levels in the placebo- and Fenfuro^®^-treated subjects

Parameters	Groups	Baseline	12-weeks of treatment	*P*-value (between groups)
C-Peptide	Placebo	3.89 ± 1.49	2.34 ± 1.77	0.0001 (Baseline)
	Fenfuro^®^	1.91 ± 2.13	1.8 ± 1.55	0.146 (12 weeks)
Thyroid Stimulating Hormone (TSH)	Placebo	9.17 ± 31.51	5.02 ± 11.37	0.188 (Baseline)
	Fenfuro^®^	2.70 ± 1.87	4.23 ± 5.30	0.688 (12 weeks)

Data are expressed as mean ± SD. *Significant reduction. Unpaired t-test was conducted to evaluate the statistical significance of data between groups.

### Effect of Fenfuro^®^ on the clinical biochemistry and immuno-hematological parameters

No significant changes in the liver, kidney, and cardiovascular function tests (SGOT, SGPT, serum bilirubin, blood urea nitrogen, creatinine, and hemoglobin levels) were observed following completion of 12-weeks of supplementation in both placebo and Fenfuro^®^ supplemented groups (Table 6). Significant reductions in the ALP level and total leukocyte count were observed; however, both the reduced values remained within the specified permissible range. Minor significant changes were observed in neutrophil, lymphocyte, and monocyte counts, but all values remained within the specified limit.

**Table 6:** Influence of placebo and Fenfuro^®^ on total blood chemistry and immuno-hematological parameters

Parameters	Groups	Baseline	12-weeks of treatment	*P*-value (between groups)
SGOT (AST) U/L)	Placebo	23.79 ± 10.36	25.78 ± 10.08	0.754
	Fenfuro^®^	23.11 ± 8.70	24.95 ± 7.77	0.675
SGPT (ALT) (U/L)	Placebo	32.20 ± 36.22	38.02 ± 22.11	0.153
	Fenfuro^®^	23.83 ± 9.82	30.64 ± 16.99	0.096
ALP (U/L)	Placebo	114.61 ± 38.33	111.55 ± 26.20	0.288
	Fenfuro^®^	106.54 ± 29.28	88.90 ± 29.44	0.001*
Serum bilirubin U/L)	Placebo	0.48 ± 0.21	0.50 ± 0.17	0.988
	Fenfuro^®^	0.54 ± 0.19	0.50 ± 0.25	0.189
Blood urea Nitrogen (mg/dL)	Placebo	31.07 ± 9.48	25.60 ± 19.49	0.845
	Fenfuro^®^	30.30 ± 12.64	26.23 ± 7.52	0.759
Creatinine (mg/dL)	Placebo	0.81 ± 0.22	0.93 ± 0.29	0.110
	Fenfuro^®^	0.93 ± 0.38	0.87 ± 0.22	0.336
Hemoglobin (g/dL)	Placebo	13.24 ± 1.6	12.69 ± 1.95	0.871
	Fenfuro^®^	13.19 ± 1.61	12.29 ± 1.8	0.346
Total leukocyte count	Placebo	8520.51 ± 3088.70	7331.57 ± 1911.86	0.088
	Fenfuro^®^	7542.85 ± 1903.93	6438.09 ± 1722.44	0.031*
Neutrophil count	Placebo	64.53 ± 8.63	63.31 ± 9.12	0.079
	Fenfuro^®^	60.11 ± 7.86	60.02 ± 7.38	0.018*
Lymphocyte count	Placebo	28.61 ± 9.16	29.00 ± 7.96	0.02*
	Fenfuro^®^	31.83 ± 7.91	32.85 ± 6.55	0.094
Monocyte count	Placebo	3.28 ± 1.23	4.10 ± 1.89	0.013*
	Fenfuro^®^	4.11 ± 1.68	3.90 ± 1.66	0.615
Eosinophil count	Placebo	3.56 ± 4.54	3.36 ± 3.23	0.803
	Fenfuro^®^	4.47 ± 5.04	3.21 ± 2.24	0.396

Data are expressed as mean ± SD. Unpaired t-test was conducted to evaluate the statistical significance of data between groups.

### Adverse events

No significant adverse events were reported in this investigation.

## Discussion

T2DM is a complex multifactorial disease controlled by several signaling pathways (38). Therefore, it has been an uphill task for clinicians and scientists to come up with an efficient therapeutic remedy against the disease. Central to the disease onset lies the insulin signaling pathway which is triggered by insulin binding mediated autophosphorylation and activation of Insulin Receptor Substrate (IRS). However, the corresponding downstream pathways are many and interconnected with crosstalks with a huge number of other cellular proteins involved in key functions. (39, 40) making the management of insulin resistance and T2DM all the more difficult.

**Fig. 5:**
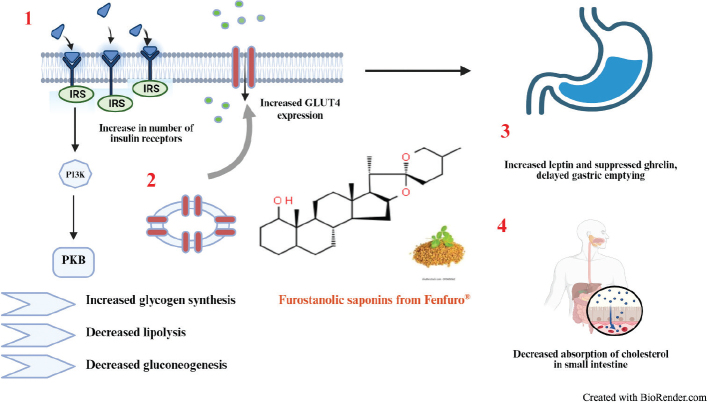
Saponin-mediated anti-diabetic effector mechanisms. These steroidal aglycones are believed to exert their action through 1) mediating increase in the number of insulin receptors causing enhanced and unabated operation of the insulin signaling pathway resulting in a variety of effector responses including increased glycogen synthesis, decreased lipolysis and gluconeogenesis; 2) upregulating GLUT4 expression for enhanced glucose uptake; and 3) decreasing hunger sensation by modulating expression of leptin and ghrelin, in addition, the mucilaginous fibrous component restricts cholesterol absorption in the small intestine.

In view of the growing concern arising out of use of conventional allopathic drugs, people affected with chronic diabetes are resorting to the use of herbal therapeutics to effectively control the rise in blood sugar and arrest development of T2DM (41, 42). Fenugreek with its optimal natural multicomponent blend of proteins, soluble mucilaginous fibers, dietary fibers, steroidal saponins, and alkaloids constitute a versatile health-promoting phytotherapeutic regime with proven effect on management of diabetes as confirmed by several studies. Gaddam et al. reported successful arrest of prediabetic persons aged between 30 and 70 years from development of diabetes on consumption of 10 g fenugreek powder before meal which successfully decreased the levels of fasting and postprandial glucose and also brought a significant reduction in the levels of low density lipoprotein (43). Kiss et al. demonstrated the insulin sensitizing effect of fenugreek seeds in a cohort group by using melanin concentrating hormone (MCH) as the indicator; improvement in insulin sensitivity was found to be inversely correlated to MCH levels (44). A recent meta-analysis conducted by Shabil et al. also revealed the potential of fenugreek seeds in alleviating hyperglycemia (45). The biological activity of fenugreek is mediated by its rich content of furostanolic saponins (46). A blend of such bioactive saponins including gallic acid, methyl gallate, and quercetin was able to successfully bring down blood glucose levels in diabetic rats by upregulation of GLUT4 receptor via AMPK dependent signaling pathway (47). Fenfuro^®^ is a novel patented formulation of our lab containing more than 45% w/w of furostanolic saponins (48) which can effectively improve insulin sensitivity and normalize blood sugar levels by a combination of mechanisms including increase in number of insulin receptors (49), delaying the gastric emptying rate thus displaying euglycemic effect by bringing down insulin requirement (50) and increasing extrahepatic utilization of insulin (20). In addition, the high content of both dietary as well as soluble mucilaginous fiber minimizes cholesterol absorption from the small intestine (51) (Fig. 5). The present study established the role of Fenfuro® as an effective phytotherapeutic to achieve significant reduction in both fasting and post-prandial glucose with concomitant decrease in % glycation of hemoglobin. The absence of any adverse side effect of the compound was confirmed by C-peptide levels which did not change significantly as well as through assessment of immunohematological parameters which also did not reveal any gross alteration. However, there was a marginal increase in levels of TSH, although within clinical range. Several studies have been conducted so far to search for the fenugreek genera with the maximum content of active ingredients (52). Although acid hydrolysis can substantially improve the yield of saponin-rich extracts from this plant, it renders subsequent development of orally administrative therapeutics challenging (53). In similar studies conducted with fenugreek seed extract alone, anti-diabetic effect was visible at a much higher dosage. In the clinical study on prediabetic patients conducted by Gaddam et al., daily intake of 10 g fenugreek seed powder for a period of 3 years resulted in significant reduction of both fasting as well as post-prandial blood glucose with parallel decrease in LDL levels (43). In another study on patients affected with Type 2 diabetes, the antidiabetic effect was barely visible with daily administration of 2 g fenugreek seed extract for 12 weeks, but there was a significant increase in insulin levels (29). Although the extracts reported in the above cases were used as sole medications without any traditional anti-diabetic medicine, the fact that Fenfuro^®^ augmented the effect of Metformin significantly within such a small amount of time and that too at a much lesser daily intake level was probably attributable to its higher content of furostanolic saponins as compared to other such preparations. Having established the beneficial effect of Fenfuro^®^ in combination with Metformin/Sulphonylurea, our next objective will be to assess its anti-diabetic effect exclusively without any supplementation. (54)

## Conclusion

Although the potential of Fenugreek has been well-acclaimed through centuries, the bioavailability of its functional components especially the furostanolic saponins has remained to be a significant bottleneck for developing effective therapeutic formulations from this plant. Fenugreek seed extract has been reported to interact with the hypothalamic–pituitary–thyroid axis by decreasing the physiological levels of the fat-regulating hormone, leptin. Therefore, the only word of caution regarding the safe administration of Fenfuro^®^ can be its restricted use in patients with hypothyroidism. Fenfuro^®^ prepared using a novel patented extraction technology from Fenugreek seeds retains not only the maximal amount of the bioactive saponins and alkaloids, but is additionally fortified with the goodness of protein, carbohydrate, and dietary fibers which not only provides relief to chronic hyperglycemia but at the same time restores overall body homeostasis. We are currently investigating the mode of intervention of Fenugreek in affecting the function of the pituitary gland with consequent imbalances in the levels of TSH and leptin. The therapeutic efficiency of Fenfuro^®^ is believed to be significantly augmented if its associated side effect can be effectively negated.

## Conflict of interest and funding

No potential conflict of interest was reported by the author(s). Partially funded by Chemical Resources, Panchkula, Haryana, India.
